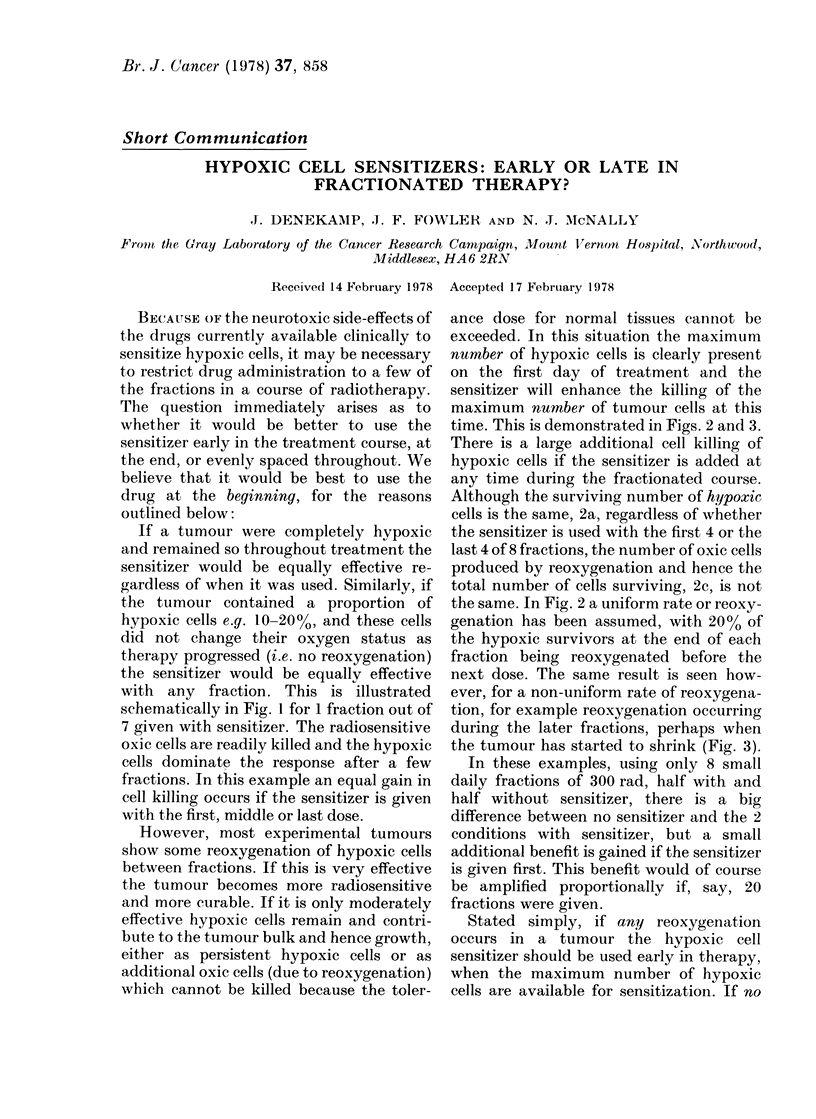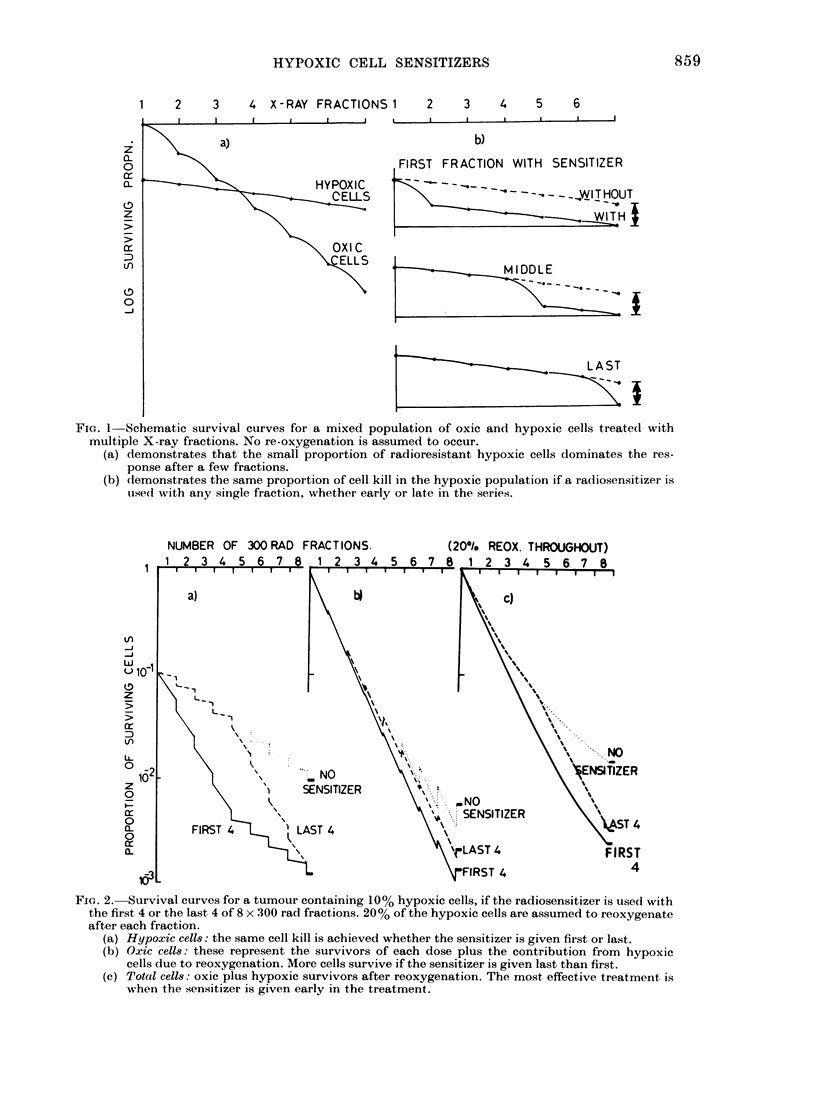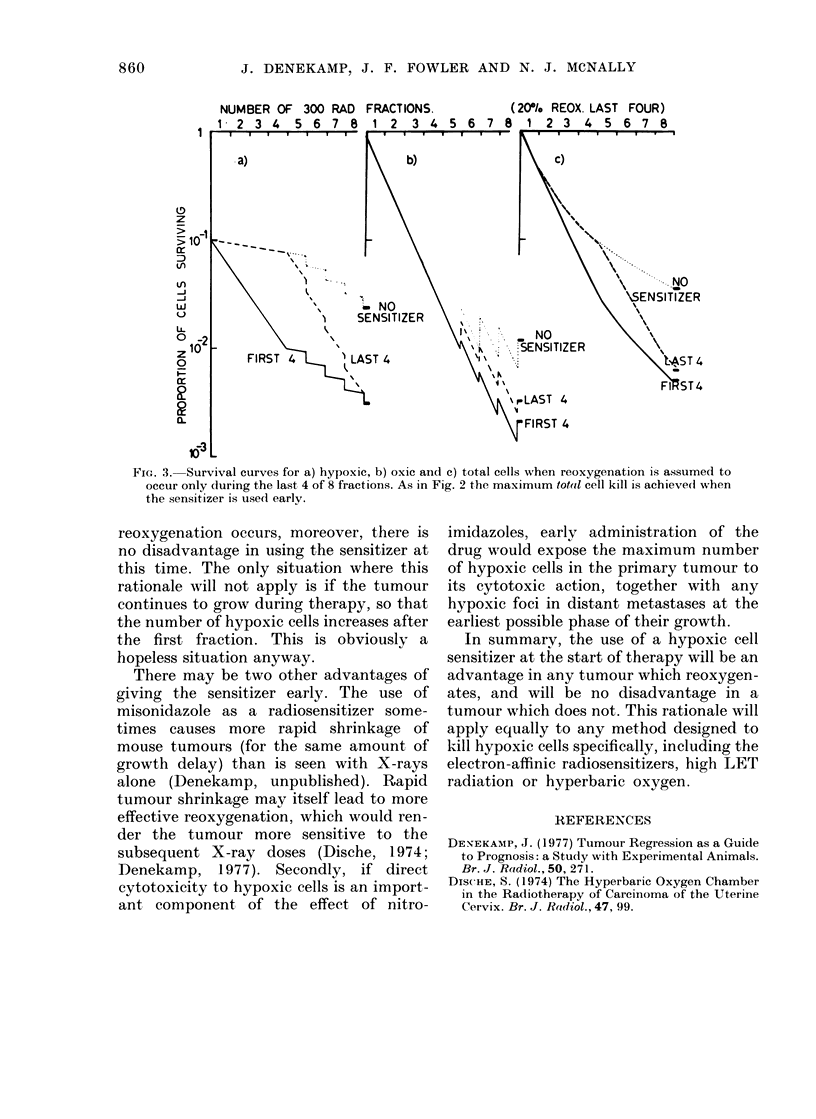# Hypoxic cell sensitizers: early or late in fractionated therapy?

**DOI:** 10.1038/bjc.1978.125

**Published:** 1978-05

**Authors:** J. Denekamp, J. F. Fowler, N. J. McNally


*Br. J. Cancer* (1978) **37**, 858

### *Short Communication*

### HYPOXIC CELL SENSITIZERS: EARLY OR LATE IN FRACTIONATED THERAPY?

J. DENEKAMP, J. F. FOWLER AND N. J. McNALLY

*From the Gray Laboratory of the Cancer Research Campaign, Mount Vernon Hospital, Northwood, Middlesex, HA6 2RN*

Received 14 February 1978 Accepted 17 February 1978

BECAUSE OF the neurotoxic side-effects of the drugs currently available clinically to sensitize hypoxic cells, it may be necessary to restrict drug administration to a few of the fractions in a course of radiotherapy. The question immediately arises as to whether it would be better to use the sensitizer early in the treatment course, at the end, or evenly spaced throughout. We believe that it would be best to use the drug at the *beginning*, for the reasons outlined below:

If a tumour were completely hypoxic and remained so throughout treatment the sensitizer would be equally effective regardless of when it was used. Similarly, if the tumour contained a proportion of hypoxic cells *e.g.* 10–20%, and these cells did not change their oxygen status as therapy progressed (*i.e.* no reoxygenation) the sensitizer would be equally effective with any fraction. This is illustrated schematically in Fig. 1 for 1 fraction out of 7 given with sensitizer. The radiosensitive oxic cells are readily killed and the hypoxic cells dominate the response after a few fractions. In this example an equal gain in cell killing occurs if the sensitizer is given with the first, middle or last dose.

However, most experimental tumours show some reoxygenation of hypoxic cells between fractions. If this is very effective the tumour becomes more radiosensitive and more curable. If it is only moderately effective hypoxic cells remain and contribute to the tumour bulk and hence growth, either as persistent hypoxic cells or as additional oxic cells (due to reoxygenation) which cannot be killed because the tolerance dose for normal tissues cannot be exceeded. In this situation the maximum *number* of hypoxic cells is clearly present on the first day of treatment and the sensitizer will enhance the killing of the maximum *number* of tumour cells at this time. This is demonstrated in Figs. 2 and 3. There is a large additional cell killing of hypoxic cells if the sensitizer is added at any time during the fractionated course. Although the surviving number of *hypoxic* cells is the same, 2a, regardless of whether the sensitizer is used with the first 4 or the last 4 of 8 fractions, the number of oxic cells produced by reoxygenation and hence the total number of cells surviving, 2c, is not the same. In Fig. 2 a uniform rate or reoxygenation has been assumed, with 20% of the hypoxic survivors at the end of each fraction being reoxygenated before the next dose. The same result is seen however, for a non-uniform rate of reoxygenation, for example reoxygenation occurring during the later fractions, perhaps when the tumour has started to shrink (Fig. 3).

In these examples, using only 8 small daily fractions of 300 rad, half with and half without sensitizer, there is a big difference between no sensitizer and the 2 conditions with sensitizer, but a small additional benefit is gained if the sensitizer is given first. This benefit would of course be amplified proportionally if, say, 20 fractions were given.

Stated simply, if *any* reoxygenation occurs in a tumour the hypoxic cell sensitizer should be used early in therapy, when the maximum number of hypoxic cells are available for sensitization. If *no*

FIG. 1—Schematic survival curves for a mixed population of oxic and hypoxic cells treated with multiple X-ray fractions. No re-oxygenation is assumed to occur.

(a) demonstrates that the small proportion of radioresistant hypoxic cells dominates the response after a few fractions.

(b) demonstrates the same proportion of cell kill in the hypoxic population if a radiosensitizer is used with any single fraction, whether early or late in the series.

FIG. 2.—Survival curves for a tumour containing 10% hypoxic cells, if the radiosensitizer is used with the first 4 or the last 4 of 8 × 300 rad fractions. 20% of the hypoxic cells are assumed to reoxygenate after each fraction.

(a) *Hypoxic cells:* the same cell kill is achieved whether the sensitizer is given first or last.

(b) *Oxic cells:* these represent the survivors of each dose plus the contribution from hypoxic cells due to reoxygenation. More cells survive if the sensitizer is given last than first.

(c) *Total cells:* oxic plus hypoxic survivors after reoxygenation. The most effective treatment is when the sensitizer is given early in the treatment.

FIG. 3.—Survival curves for a) hypoxic, b) oxic and c) total cells when reoxygenation is assumed to occur only during the last 4 of 8 fractions. As in Fig. 2 the maximum *total* cell kill is achieved when the sensitizer is used early.

reoxygenation occurs, moreover, there is no disadvantage in using the sensitizer at this time. The only situation where this rationale will not apply is if the tumour continues to grow during therapy, so that the number of hypoxic cells increases after the first fraction. This is obviously a hopeless situation anyway.

There may be two other advantages of giving the sensitizer early. The use of misonidazole as a radiosensitizer sometimes causes more rapid shrinkage of mouse tumours (for the same amount of growth delay) than is seen with X-rays alone (Denekamp, unpublished). Rapid tumour shrinkage may itself lead to more effective reoxygenation, which would render the tumour more sensitive to the subsequent X-ray doses (Dische, 1974; Denekamp, 1977). Secondly, if direct cytotoxicity to hypoxic cells is an important component of the effect of nitroimidazoles, early administration of the drug would expose the maximum number of hypoxic cells in the primary tumour to its cytotoxic action, together with any hypoxic foci in distant metastases at the earliest possible phase of their growth.

In summary, the use of a hypoxic cell sensitizer at the start of therapy will be an advantage in any tumour which reoxygenates, and will be no disadvantage in a tumour which does not. This rationale will apply equally to any method designed to kill hypoxic cells specifically, including the electron-affinic radiosensitizers, high LET radiation or hyperbaric oxygen.

### REFERENCES

DENEKAMP, J. (1977) Tumour Regression as a Guide to Prognosis: a Study with Experimental Animals. *Br. J. Radiol.*, **50**, 271.

DISCHE, S. (1974) The Hyperbaric Oxygen Chamber in the Radiotherapy of Carcinoma of the Uterine Cervix. *Br. J. Radiol.*, **47**, 99.